# Are higher antibody levels against seasonal human coronaviruses associated with a more robust humoral immune response after SARS-CoV-2 vaccination?

**DOI:** 10.3389/fimmu.2022.954093

**Published:** 2022-09-08

**Authors:** Michael Asamoah-Boaheng, Brian Grunau, Mohammad Ehsanul Karim, Agatha N. Jassem, Jennifer Bolster, Ana Citlali Marquez, Frank X. Scheuermeyer, David M. Goldfarb

**Affiliations:** ^1^ Department of Emergency Medicine, University of British Columbia, Vancouver, BC, Canada; ^2^ Faculty of Medicine, Clinical Epidemiology, Memorial University of Newfoundland, St John’s, NL, Canada; ^3^ Centre for Health Evaluation & Outcome Sciences, University of British Columbia, Vancouver, BC, Canada; ^4^ Clinical and Medical Programs, British Columbia Emergency Health Services, Vancouver, BC, Canada; ^5^ School of Population and Public Health, University of British Columbia, Vancouver, BC, Canada; ^6^ Department of Pathology and Laboratory Medicine, University of British Columbia, Vancouver, BC, Canada; ^7^ Public Health Laboratory, British Columbia Centre for Disease Control, Vancouver, BC, Canada; ^8^ British Columbia Children’s Hospital Research Institute, British Columbia Children’s Hospital, Vancouver, BC, Canada

**Keywords:** human endemic coronaviruses, SARS-COV-2 antibodies, vaccination, COVID-19, antibody concentrations

## Abstract

The SARS-CoV-2 belongs to the coronavirus family, which also includes common endemic coronaviruses (HCoVs). We hypothesized that immunity to HCoVs would be associated with stronger immunogenicity from SARS-CoV-2 vaccines. The study included samples from the COSRIP observational cohort study of adult paramedics in Canada. Participants provided blood samples, questionnaire data, and results of COVID-19 testing. Samples were tested for anti-spike IgG against SARS-CoV-2, HCoV-229E, HCoV-HKU1, HCoV-NL63, and HCoV-OC43 antigens. We first compared samples from vaccinated and unvaccinated participants, to determine which HCoV antibodies were affected by vaccination. We created scatter plots and performed correlation analysis to estimate the extent of the linear relationship between HCoVs and SARS-CoV-2 anti-spike antibodies. Further, using adjusted log-log multiple regression, we modeled the association between each strain of HCoV and SARS-CoV-2 antibodies. Of 1510 participants (mean age of 39 years), 94 (6.2%) had a history of COVID-19. There were significant differences between vaccinated and unvaccinated participant in anti-spike antibodies to HCoV-HKU1, and HCoV-OC43; however, levels for HCoV-229E and HCoV-NL63 were similar (suggesting that vaccination did not affect these baseline values). Among vaccinated individuals without prior COVID-19 infection, SARS-COV-2 anti-spike IgG demonstrated a weak positive relationship between both HCoV-229E (r = 0.11) and HCoV-NL63 (r = 0.12). From the adjusted log-log multiple regression model, higher HCoV-229E and HCoV-NL63 anti-spike IgG antibodies were associated with increased SARS-COV-2 anti-spike IgG antibodies. Vaccination appears to result in measurable increases in HCoV-HKU1, and HCoV-OC43 IgG levels. Anti-HCoV-229E and HCoV-NL63 antibodies were unaffected by vaccination, and higher levels were associated with significantly higher COVID-19 vaccine-induced SARS-COV-2 antibodies.

## Introduction

Severe acute respiratory syndrome coronavirus 2 (SARS-CoV-2), a betacoronavirus in the sarbecovirus family, has caused significant global morbidity and mortality. Vaccines have been introduced which generate robust immune responses against SARS-CoV-2 and also provide effective protection particularly against the development of severe disease (1, 2). Previous studies have also shown that COVID-19 infections also result in significant protection against subsequent SARS-CoV-2 re-infection (3–5). However, there are likely other processes which impact an individual’s immune protection to SARS-COV-2, including past exposures and pre-existing immunity to other similar viruses (1, 6, 7).

Serological studies have documented evidence of antibodies to other human endemic coronaviruses (HCoV’s), which include leading causes of the “common cold” (8). Given the similarities between SARS-CoV-2 and HCoV’s, and that exposure to HCoV’s in the general population is common (at least prior to nonpharmaceutical interventions implemented during the COVID-19 pandemic), investigations into the potential correlation of HCoV’s and SARS-COV-2 antibodies (through vaccination or previous COVID-19 infection) may provide further insight into their contribution to SARS-COV-2 immunity (1, 9–11).

From a cohort of vaccinated and unvaccinated individuals, we investigated serum cross-reactivity between anti-spike IgG antibodies against SARS-CoV-2 and four common endemic coronaviruses (the alpha coronaviruses HCoV-229E and HCoV-NL63, and the beta coronaviruses, HCoV-HKU1, HCoV-OC43), hereafter collectively referred to as “HCoV’s”. We hypothesized that individuals with higher baseline HCoV antibody serum reactivity would exhibit a higher immune response against SARS-CoV-2 spike after vaccination. First, we sought to compare vaccinated to unvaccinated samples, to identify which HCoV antibody levels were affected by vaccination. Among those unaffected by vaccination, we investigated the association of the various HCoV anti-spike IgG levels with SARS-CoV-2 anti-spike IgG levels, to determine if HCoV immunogenicity appears to enhance the SARS-CoV-2 immune response. Second, among those HCoV for which antibodies were affected by vaccination, we explored the correlation between post-vaccination HCoV and SARS-CoV-2 antibody levels.

## Methods

### Study design and participants

We used samples from the COVID-19 Occupational Risks, Seroprevalence and Immunity among Paramedics in Canada (CORSIP) study, a prospective cohort study designed to investigate the seroprevalence of SARS-CoV-2 and also immune responses in this group of front-line health care workers. Participants provided blood samples upon enrollment, questionnaire data, and results of all SARS-CoV-2 polymerase chain reaction (PCR) and rapid antigen tests and vaccinations. We included all samples that had complete data on antibody testing irrespective of their vaccination and COVID-19 status. Participants with either a previous positive COVID-19 PCR or rapid antigen test or a reactive Nucleocapsid Elecsys Anti-SARS-Cov-2 test were considered to have previously been infected with SARS-CoV-2.

### Serological testing

We tested all samples with: the Roche Nucleocapsid Elecsys Anti-SARS-Cov-2 assay; and, the Meso scale discovery (MSD) V-PLEX COVID-19 Coronavirus Panel 2 IgG assay, which measures IgG antibodies to the SARS-CoV-2 spike (S1), receptor binding domain (RBD), spike N-terminal domain (NTD), and nucleocapsid (N) antigens, as well as to SARS-CoV-1 Spike, HCoV-229E Spike, HCoV-HKU1 Spike, HCoV-NL63 Spike, and HCoV-OC43 Spike antigens.

### Statistical analysis

We sequentially classified participants as: (i) COVID-19 positive; (ii) COVID-19 negative and vaccinated (provided they had received ≥ 1 dose); or, (iii) COVID-19 negative and unvaccinated. We compared participant characteristics, and SARS-CoV-2 and HCoV antibody concentrations between vaccinated and unvaccinated COVID-19 negative cases with a Mann Whitney U-test, in order to identify which HCoV results were likely affected by vaccination (and thus we could not assume these values represented the pre-vaccine antibody concentrations). Similar statistical tests were conducted to compare participant characteristics between unvaccinated cases and those with prior COVID-19. Among COVID-19-negative individuals, we used scatter plots to illustrate graphically the relationship between the HCoVs and SARS-Cov-2 spike antibodies among participants. Using these same groups, we estimated the Pearson correlation coefficient (**r**) with associated ***p*** *-*values to measure the magnitude of the relationship between the HCoVs and SARS CoV-2 antibody concentration. Among vaccinated COVID-negative cases, we used multiple log-log linear regression to investigate the association of HCoV concentrations with SARS-CoV-2 spike antibody concentrations, after adjusting for sex, age, education, ‘interval (days) from first vaccine dose to blood collection’, ‘interval (days) from last vaccine to blood collection’ and ‘interval (days) from January 1, 2021 to blood collection’.

## Results

A total of 1510 participants were eligible for this study, of whom 94 (6.2%) had a history of COVID-19 (69 of whom were vaccinated, and 25 unvaccinated); of COVID-19 negative participants (n=1416), 1240 (88%) were vaccinated (988 with BNT162b2 and 226 with mRNA-1273 vaccine) and 176 (12%) were unvaccinated. Participant characteristics, stratified by COVID-19 and vaccination status, are shown in **Table 1**. Overall, the mean age was 39 years with 640 (42%) reporting female sex at birth, without significant differences between subgroups. Among samples from participants without previous COVID-19, there were significant differences in anti-spike antibody concentrations between vaccinated and unvaccinated groups for HCoV-HKU1, and HCoV-OC43, suggesting that vaccination affected these baseline values. Values for HCoV-229E and HCoV-NL63 were similar, suggesting that vaccination did not affect baseline values.

**Table 1:** Participant characteristics by vaccination status.

	Overall	COVID-Negative (n=1416)				COVID-Positive (n=96)	

Participant’s characteristics	N=1510 (%)	Vaccinated, n =1240 (88%)	Unvaccinated, n =176 (12%)	*P*-value (Vacc vrs unvacc)	Positive COVID-19, N=94 (6%)		*P-*value(Unvaccinated vs. COVID-19 +)
Age [mean (SD)]	39 (10)	39 (11)	39 (10)	0.544	39 (10)		0.935
*Sex (at birth)*							
Male, n (%)	812 (54)	677 (55)	90 (51)		48 (51)		0.731
Female, n (%)	640 (42)	515 (42)	84 (48)	0.257	41 (44)		0.731
Prefer not to answer, n (%)	58 (4.0)	48 (4.0)	2.0 (1.0)			5.0 (5.0)
*Educational level*							
Non-university certificate/diploma	955 (63)	760 (61)	131 (74)		64 (68)		
University bachelor’s degree	445 (30)	382 (31)	40 (23)		23 (25)		
Masters or Doctorate	45 (3.0)	42 (3.0)	2.0 (1.0)	0.019	1.0 (1.0)		0.962
Others	65 (4.0)	56 (5.0)	3.0 (2.0)		6.0 (6.0)		
January 1 to blood collection date [*median (IQR)*]	102 (62, 147)	104 (63, 152)	87 (56, 117)	0.000	107 (63, 154)		0.045
*Vaccination type*							
BNT162b2	1042 (69)	986 (80)	n/a	n/a	56(81)		n/a
mRNA-1273	235 (16)	226 (18)	n/a	n/a	9.0 (13)		n/a
Others	1.0 (0.0)				1.0 (1.4)		n/a
Did not answer	31 (2.0)	28 (2.0)	n/a	n/a	3.0 (4.3)		n/a
*Vaccine Doses*							
1^st^ & 2^nd^ Doses (BNT162b2)	894 (59)	842 (68)	n/a	n/a	52 (55)		n/a
1^st^ & 2^nd^ Doses (mRNA-1273)	187 (12)	179 (14)	n/a	n/a	8.0 (8.5)		n/a
Only one vaccine (BNT162b2)	134 (8.9)	131 (11)	n/a	n/a	3.0 (3.19)		n/a
Only one vaccine (mRNA-1273)	37 (2.5)	37 (3.0)	n/a	n/a	0.0 (0.0)		n/a
Others	1.0 (0.1)	0.0 (0.0)	n/a		0.0 (0.0)		n/a
1^st^ vaccine to blood collection date [median (IQR)]	84 (47, 124)	84 (47, 125)	n/a	n/a	90 (54, 111)		n/a
2^nd^ Vaccine to blood collection [median (IQR)]	65 (34, 108)	65 (34, 108)	n/a	n/a	64 (35, 89)		n/a
*Endemic coronaviruses* [gMean (gSD)]							
HCoV-229E Spike	19699 (2.0)	19351 (2.0)	21249 (2.0)	0.687	21647 (2.0)		0.800
HCoV-HKU1 Spike	11963(3.0)	12488 (3.0)	8420 (3.0)	0.000	13130 (3.0)		0.001
HCoV-NL63 Spike	3843 (3.0)	3892 (3.0)	3613 (2.0)	0.573	3644 (2.0)		0.598
HCoV-OC43 Spike	40142 (2.0)	41274 (2.0)	27885 (2.0)	0.000	55270 (2.0)		0.000
*SARS-CoV-2 antibodies* [gMean (gSD)]							
MSD SARS-CoV-2 N	204 (5.0)	172 (4.0)	139 (4.0)	0.260	4045 (10)		0.000
MSD SARS-CoV-2 NTD	279 (14.0)	486 (9.0)	4 (4.0)	0.000	577 (10)		0.000
MSD SARS-CoV-2 RBD	12027 (12)	20139 (8.0)	257 (4.0)	0.000	18366 (10)		0.000
MSD SARS-CoV-2 Spike	15577 (18)	29822 (10)	96 (5.0)	0.000	31815 (10)		0.000

gMean, geometric mean; gSD, geometric standard deviation; SD, Standard deviation; IQR, interquartile range; n/a, not applicable.

**Figure 1:**
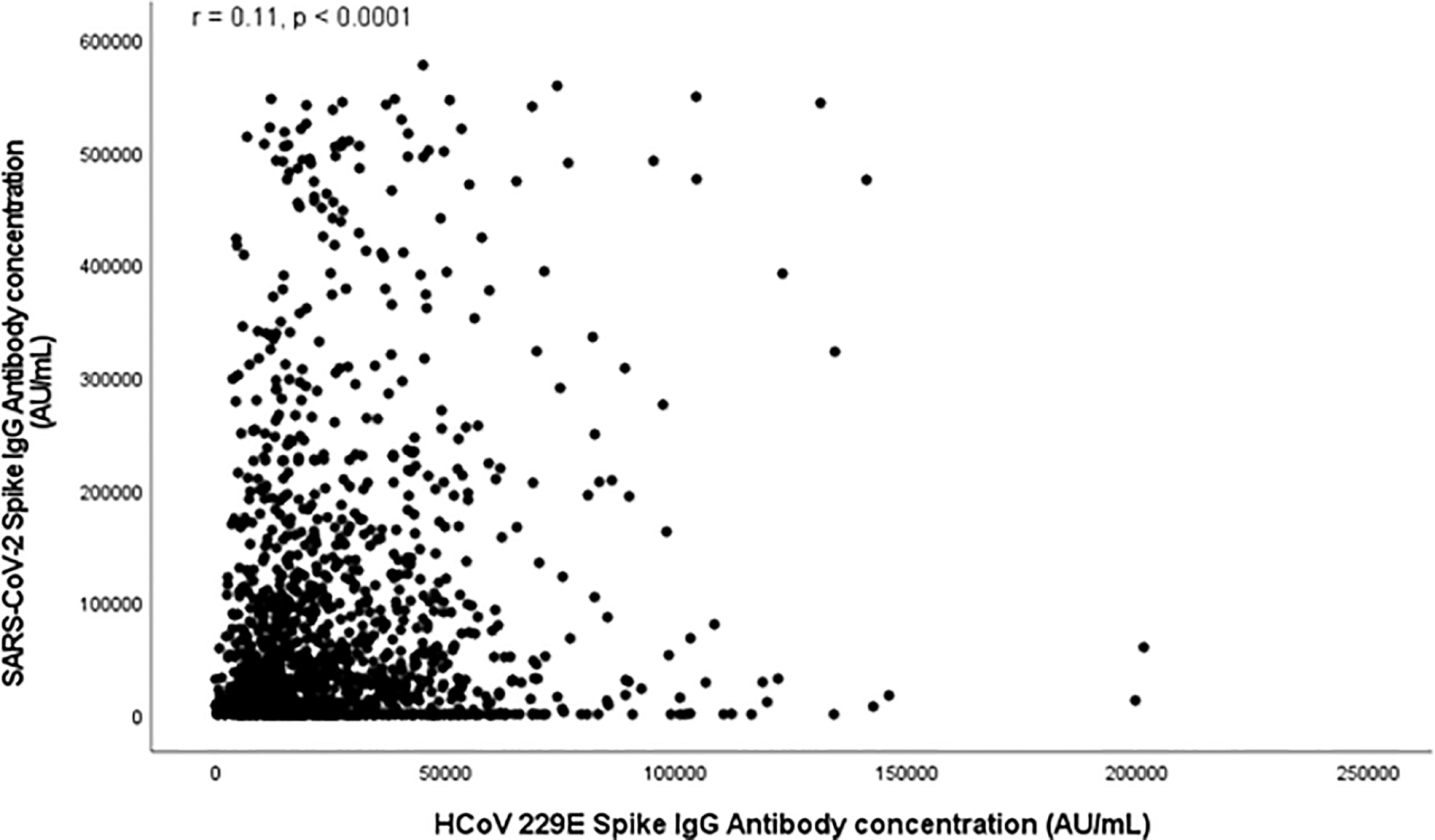
Scatter plot depicting relationship between SARS-CoV-2 Spike Antibody Concentration (AU/mL) and HCoV 229E Spike antibodies (AU/mL).

**Figure 2:**
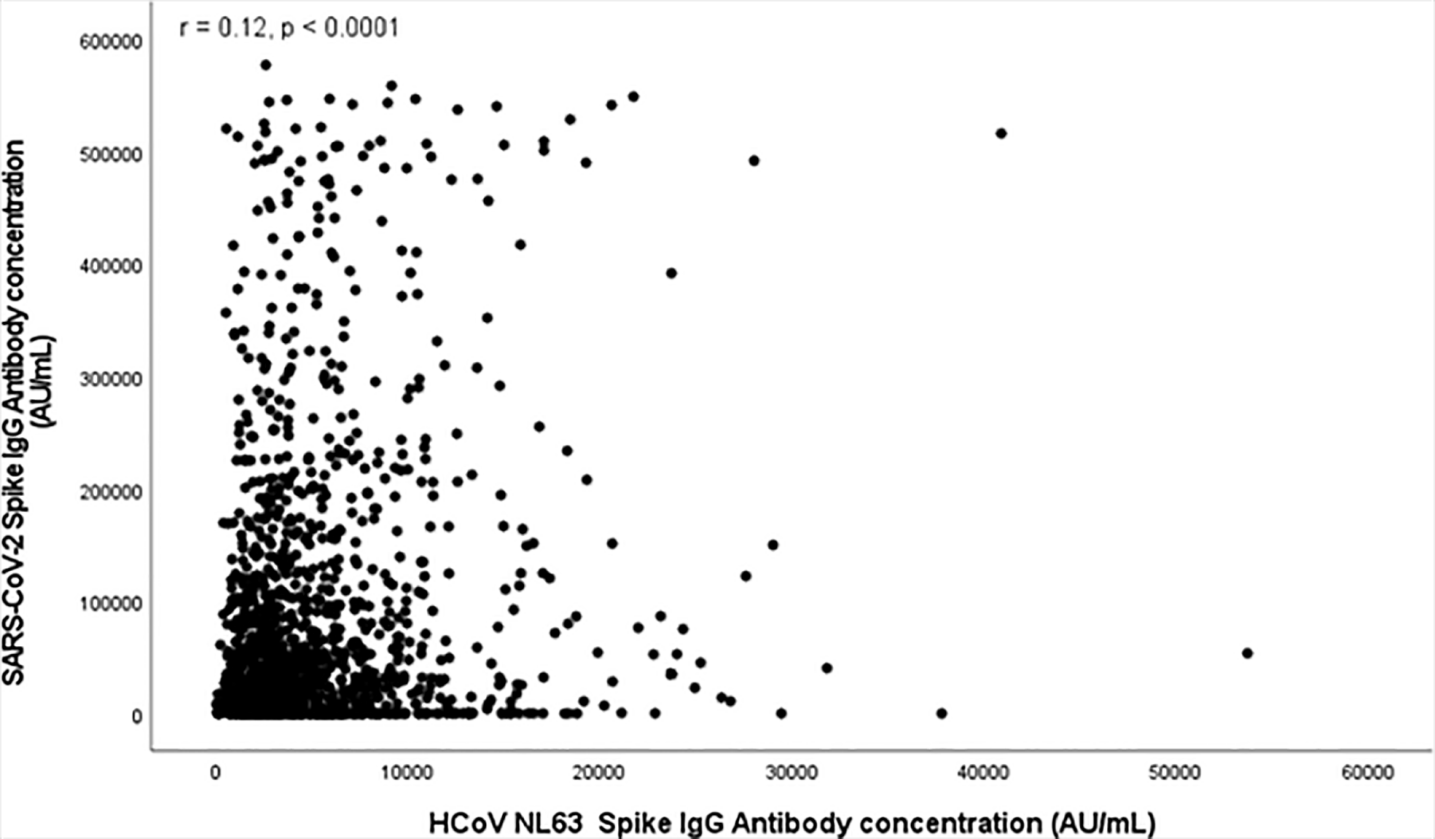
Scatter plot depicting relationship between SARS-CoV-2 Spike Antibody Concentration (AU/mL) and HCoV NL63 Spike antibodies (AU/mL).

**Figures 1**, **2** and **S1-S2** (in the **supplementary material**) show scatter plots illustrating the relationship between HCoV and SARS-Cov-2 spike antibody concentrations. HCoV-229E (**r** = 0.11, *p* < 0.0001) and HCoV-NL63 (r = 0.12, *p* < 0.0001) spike antibody concentrations both demonstrated weak positive correlation with SARS-Cov-2 spike antibody concentrations. HCoV-HKU1 (*r* = 0.26) and HCoV-OC43 (*r* = 0.31) also demonstrated positive linear relationship with SARS-CoV-2.

Results of the multivariate log-log multiple linear regression model among the vaccinated COVID-19 negative participants are shown in **Table 2**. From the results, each unit increase in the HCoV-229E and HCoV-NL63 antibody concentrations were significantly associated with increased SARS-COV-2 antibody concentrations. The adjusted mean log SARS-CoV-2 IgG antibody concentration increased by 0.17 when the antibodies against HCoV increased. Similarly, the adjusted mean log SARS-CoV-2 antibody concentration among vaccinated participants increased by 0.18 when the antibodies against HCoV-NL63 increased. We found similar results for the other two HCoV’s.

**Table 2:** Multivariate analysis of cross-reactivity between exposure to endemic coronaviruses and SARS-CoV-2 antibody concentration among vaccinated participants.

Model types	Variables	β (95% CI)
Model 1	HCoV-229E Spike	0.17(0.04, 0.30) ***
Model 2	HCoV-HKU1 Spike	0.56(0.44, 0.69) ***
Model 3	HCoV-NL63 Spike	0.18(0.05, 0.32) ***
Model 4	HCoV-OC43 Spike	0.70(0.55, 0.84) ***

Outcome variable: SARS-CoV-2 Anti-spike antibody concentration

Each model was adjusted for age, sex, education, and “days from January to blood collection”, date from 1^st^ vaccine to blood collection, and date from 2^nd^ vaccine to blood collection,

***p < 0.05, i.e., variable in the model is significant at (p<0.05).

β (95% CI): Regression coefficients (95% Confidence interval).

## Discussion

We examined cross-reactivity between immunogenicity to four endemic coronaviruses with SARS-CoV-2 anti-spike antibodies among over 1500 participants. First, we found that among COVID-19-negative participants, HCoV-HKU1 and HCoV-OC43 anti-spike antibody concentrations were significantly different between vaccinated and unvaccinated cases, suggesting that antibodies generated by the SARS-CoV-2 vaccine significantly react with HCoV spike from these betacoronaviruses. This may be due to the fact that all of these are beta coronaviruses that share more spike sequence homology with SARS-CoV-2 than the more distantly related alpha HCoVs (-229E and –NL63) (12). Second, higher baseline IgG levels to HCoV-229E and HCoV-NL63 were associated with a stronger vaccine-induced SARS-CoV-2 antibody response, suggesting that baseline antibody reactivity to these viruses boosts SARS-CoV-2 immune response. The impact of prior antibody reactivity to HCoV-HKU1 Spike, and HCoV-OC43 on vaccine-facilitated immune responses is unclear from our data. These data will assist future investigations by demonstrating the non-specific nature of post-vaccine antibody levels for certain HCoVs and support further research into any potential protective benefits against COVID-19 through HCoV exposure.

Our main study goal was to determine if pre-existing antibodies against HCoV would be associated with a stronger vaccine-induced SARS-CoV-2 immune response. Our data suggests that HCoV-HKU1, and HCoV-OC43 antibody levels were affected by vaccination, and thus we were unable to test this hypothesis. For HCoV-229E and HCoV-NL63, however, baseline antibody levels did not appear to be affected by vaccination. We found that antibody levels to the spike proteins of these viruses were associated with the SARS-CoV-2 anti-spike antibody levels, supporting our hypothesis.

Our study demonstrates a relationship between all HCoVs and SARS-CoV-2 antibody concentration after controlling for important study covariates. For the case of HCoV-HKU1, HCoV-OC43, while it is possible that vaccinated individuals had substantially higher previous antibody reactivity to these viruses, it is much more likely that values are affected by the SARS-CoV-2 immune response—the clinical relevance of this enhanced antibody response in terms of protection from infection remains to be determined. While these results may be a result of lab assay cross-reactivity, it is possible that SARS-CoV-2 vaccination does, in fact, enhance immunity to other coronaviruses than just SARS-CoV-2. Also, the design of this study did not allow for specific analysis of B cell responses. Others have demonstrated that B cell memory response against endemic coronaviruses may play some role in provision of cross-reactive immunity to SARS-CoV-2 (13), however this requires further study.

These results corroborate some of the emerging literature around a potential cross-reactivity between HCoVs and SARS-CoV-2 antibodies. A study by Grobben et al. (2021) (14) detected cross-reactive antibodies to all human endemic coronaviruses after SARS-CoV-2 vaccination. Also, a two-way cross-reactivity was observed between SARS-CoV-2 and HCoV-OC43 in a study that investigated and profiled temporal changes of IgG antibody against spike proteins of SARS-CoV-2 and seasonal HCoVs (15). In a recent study which investigated the impact of COVID-19 vaccination on neutralization of seasonal coronaviruses, COVID-19 vaccination resulted in efficient cross-neutralization of SARS-CoV-1 antibody concentrations (16). Pertaining to HCoV-229E Spike and HCoV-NL63, our data suggests that pre-existing immunity to HCoVs could partially explain differences in vaccine-based antibody responses and may also affect risk of COVID-19 and/or disease severity. Previous data has shown that children have overall fared well during the COVID-19 pandemic, with lower incidence and less severe disease. Preceding immunity to HCoV’s may play a role in this observation (17).

The implementation of nonpharmaceutical interventions (NPIs) as part of the pandemic response has resulted in the virtual disappearance of HCoVs from circulation in many jurisdictions, similar as to what has been seen with other respiratory viruses (18). With the lifting of NPIs in many jurisdictions globally, HCoVs will likely resume circulation. It will be important to understand what, if any, impacts re-exposure to these viruses will have with respect to SARS-CoV-2 immunity.

## Conclusion

We found significant differences in HCoV-HKU1 and HCoV-OC43 anti-spike concentrations between COVID-19-vaccinated and unvaccinated participants, suggesting assay cross-reactivity. Higher HCoV-229E and HCoV-NL63 anti-spike concentrations were associated with a higher SARS-Cov-2 antibody response, suggesting that pre-existing HCoVs immunity may bolster vaccine-induced immune response to SARS-Cov-2.

## Data availability statement

The raw data supporting the conclusions of this article will be made available by the authors, without undue reservation.

## Ethics statement

The studies involving human participants were reviewed and approved by the University of British Columbia and the University of Toronto research ethics boards. The patients/participants provided their written informed consent to participate in this study.

## Author contributions

Conceptualization: MA-B, BG, and DG; formal analysis: MA-B, BG, DG, MK, AJ, JB, AM, and FS; investigation: MA-B, BG, DG, MK, AJ, JB, AM, and FS; methodology: MA-B and BG; data analysis: MA-B; project administration: BG and DG; supervision: BG and DG; validation: MA-B, BG, DG, MK, AJ, JB, AM, and FS; writing of manuscript: MB, BG, DG, MK, AJ, JB, AM, and FS. All authors contributed to the article and approved the final version.

## Funding

This study was supported by funding from Government of Canada, through the COVID-19 Immunity Task Force. MK is supported in part by a Scholar Award from the Michael Smith Foundation for Health Research, partnered with Centre for Health Evaluation and Outcome Sciences. BG is supported by the Michael Smith Foundation for Health Research.

## Conflict of interest

The authors declare that the research was conducted in the absence of any commercial or financial relationships that could be construed as a potential conflict of interest.

## Publisher’s note

All claims expressed in this article are solely those of the authors and do not necessarily represent those of their affiliated organizations, or those of the publisher, the editors and the reviewers. Any product that may be evaluated in this article, or claim that may be made by its manufacturer, is not guaranteed or endorsed by the publisher.
