# Supplementary material for: Are higher antibody levels against seasonal human coronaviruses associated with a more robust humoral immune response after SARS-CoV-2 vaccination?
Source: Front Immunol. 2022 Sep 8;13:954093. doi: 10.3389/fimmu.2022.954093 (PMC9493031; doi:10.3389/fimmu.2022.954093)
Supplement: Supplementary file 1 [file DataSheet_1.docx]

Supplementary Material

## Supplementary Figures

**Figure S1**: Scatter plot depicting relationship between SARS-CoV-2 Spike Antibody Concentration (AU/mL) and HCoV OC43 Spike antibodies (AU/mL)

**Figure S2**: Scatter plot depicting relationship between SARS-CoV-2 Spike Antibody Concentration (AU/mL) and HCoV HKU1 Spike antibodies (AU/mL)
